# House Air—The Cause and Promotor of Disease

**Published:** 1878-05

**Authors:** 


### BIBLIOGRAPHICAL.

*House Air—the Cause and Promotor of Disease.*—A pamphlet by Frank Donaldson, M. D., Professor of Physiology and Hygiene, Maryland University, School of Medicine.

An interesting and able exposition of the injurious effects of confined air on health. Dr. Donaldson shows in this paper many facts, which, while we are all more or less familiar with, yet cannot be too often brought before the public. To dentists especially is the subject one of importance, as the ill health they so often suffer is undoubtedly due in many cases to the want of proper ventilation. It is bad enough to stand in a constrained posture, with all the attention on the stretch and every muscle and nerve in tension; but to breathe, as many do, the confined air of the office, and add to that the inhalation hour after hour of the breath of our patients, is not slow but rapid poisoning. Some one ought to invent a contrivance by which a gentle circulation, without draft enough to cause annoyance, could be kept up around the dental chair. Dr. Donaldson's paper touches on the sunshine question also, and shows that it is directly promotive of health. It is probable from the few observations we have been able to make, that not a few dentists have of late moved their chairs to the south side of the house. We would be glad to know how many have of late been influenced by the discussions of the light question, and solicit communications.

